# Reduced Fc-mediated antibody responses after COVID-19 mRNA vaccination in a cohort of people living with HIV-1

**DOI:** 10.1038/s41598-025-26149-z

**Published:** 2025-11-25

**Authors:** Jéromine Klingler, Priyanka Gadam Rao, Juan C. Bandres, Ismael Pena, Katherine Bolanos Roldan, Gagandeep Singh, Brian Monahan, Charles Gleason, Yuexing Chen, Stefan Slamanig, Weina Sun, Chitra Upadhyay, Catarina E. Hioe

**Affiliations:** 1 James J. Peters VA Medical Center, Bronx, NY USA; 2 Division of Infectious Diseases, Department of Medicine, Icahn School of Medicine at Mount Sinai, New York, NY USA; 3 Department of Microbiology, Icahn School of Medicine at Mount Sinai, New York, NY USA; 4 Center for Vaccine Research and Pandemic Preparedness, Icahn School of Medicine at Mount Sinai, New York, NY USA

**Keywords:** COVID-19, SARS-CoV-2, Vaccines, HIV-1, Antibodies, Fc activities, Diseases, Immunology, Medical research, Microbiology

## Abstract

HIV-1 infection has been associated with increased COVID-19-related hospitalization and greater SARS-CoV-2 shedding. People living with HIV-1 (PLWH) also have higher risks to other respiratory infections and lower response rates to influenza and pneumococcal vaccines, even after antiretroviral therapy. This observational study evaluated serum antibody responses after mRNA COVID-19 vaccination in elderly male cohorts of PLWH on antiretroviral therapy and people without HIV-1 (PWOH). Specifically we measured the titers, isotypes/subtypes, and functions of antibodies after the third dose of mRNA COVID-19 vaccines. SARS-CoV-2-specific total immunoglobulin (Ig) titers were significantly higher in blood samples from PLWH vs. PWOH. Notably, PLWH had higher levels of IgG2 and IgG4, two IgG subtypes with minimal Fc activities. Correspondingly, lower Fc capacities were displayed by PLWH, and they correlated inversely with SARS-CoV-2-specific IgG4 levels. Our data point to changes in SARS-CoV-2-specific antibody distribution elicited in PLWH after mRNA COVID-19 vaccinations, resulting in the curtailments in the Fc functionalities that may contribute to suboptimal antiviral responses.

**Supplementary Information:**

The online version contains supplementary material available at 10.1038/s41598-025-26149-z.

## Introduction

Most COVID-19 vaccines are designed with SARS-CoV-2 spike as the key immunogen to elicit virus-neutralizing antibodies^1^. Beyond neutralization, antibodies can also engage the innate immune system to mediate effector functions, such as complement-mediated lysis and antibody-dependent cellular cytotoxicity, phagocytosis, trogocytosis, and complement deposition (ADCC, ADCP, ADCT, ADCD, respectively)^2^. These activities are dependent on Fc engagement with Fc or complement receptors present on natural killer cells, neutrophils, macrophages, and other innate immune cells, and are dictated by antibody isotypes and subtypes. Among IgG subtypes, only IgG1 and IgG3 are effective in binding C1q to initiate the classical complement activation, while IgG2 and IgG4 are largely ineffective^3^. IgG1 and IgG3 also display the strongest affinities for Fcγ receptors whereas IgG4 has the lowest affinity^4,5^.

Repeated administration of vaccines against pertussis, HIV-1, malaria, and COVID-19 have been shown to increase vaccine-specific IgG4 responses disproportionately in the vaccine platform-dependent fashion^6^. The COVID-19 mRNA vaccines in particular have been shown to induce high levels of SARS-CoV-2-specific IgG and IgA in the blood, with IgG responses characterized with high-level IgG1, along with IgG2, IgG3, and IgG4 at lower levels^7–11^. Indeed, a broad immunoglobulin spectrum from IgM, IgG1 to IgG4, IgA1, and IgA2 is induced by COVID-19 mRNA vaccines, in contrast to the narrower isotype profile observed in the blood of convalescent COVID-19 patients^8,10^. Interestingly, subsequent boosts with mRNA vaccines have been marked by increasing IgG4 titers starting from six months after two vaccinations to eventually overtake IgG1 after the fourth dose^12–15^. Non-mRNA COVID-19 vaccine platforms that utilize adenovirus vectors, inactivated virions, and recombinant proteins have not been associated with such IgG4 shifts^6^, although low and transient induction of spike-specific IgG4 has been noted one month after the Sputnik V Ad26/Ad5 vaccinations and not at later time points^8^. Nonetheless, anti-spike antibodies generated after Ad26/Ad5 immunizations comprise mainly of IgG1 and display similar or greater Fc potencies than those elicited by mRNA vaccines, while neutralization titers against early SARS-CoV-2 variants were indistinguishable^8^.

Unlike virus neutralization, Fc activities do not directly block virus infection but can mitigate severe disease by reducing virus burden and eradicating infected cells, especially in the face of emerging variants resistant to neutralizing antibodies^16^. However, the Fc functionality of SARS-CoV-2-specific antibodies among people living with HIV-1 (PLWH), especially older male PLWH with comorbidities at risk of severe COVID-19, has not been much studied^17,18^. PLWH, even with effective antiretroviral therapy (ART), have higher SARS-CoV-2 viral loads in nasal swabs, higher rates of hospitalization, and increased risk of persistent SARS-CoV-2 infection than people without HIV-1 (PWOH)^19–22^. In a recent study, a systems immunology approach demonstrated lower humoral and B cell responses to SARS-CoV-2, as well as less coordination among SARS-CoV-2-binding antibodies, functional antibody responses, and B cell and CD4 T cell magnitudes among convalescent PLWH vs. PWOH after symptomatic outpatient COVID-19 ^23^. PLWH also exhibit impaired immune responses to several respiratory infections and respond poorly to vaccines against influenza virus, pneumococcal disease, hepatitis B virus, and human papillomavirus^24–27^. While SARS-CoV-2-specific antibodies with robust Fc functions have been shown to be elicited in response to various COVID-19 vaccine formats in PWOH^28^, much less is known about these responses in PLWH and indepth evaluations of antibody isotypes/subtypes remain lacking. A previous study demonstrated no significant differences in SARS-CoV-2 RBD-specific antibody avidity, ADCC activity, and virus neutralizing titers of PLWH with CD4 counts < 250/mm^3^ vs. > 500/mm^3^, but no comparison with PWOH was performed^29^. Other studies also showed no difference in neutralization and Fc effector functions (notably ADCC, ADCD, ADCT and ADCP) of PLWH vs. PWOH, with the majority of the study participants under 55 years old^30,31^. Comparable spike-specific antibody binding levels and neutralization activities were also detected after three doses of COVID-19 mRNA in vaccinated PLWH and PWOH who were solid organ transplant recipients, but Fc profiles and activities were not studied^32^. Of note, a disproportionnal increase in SARS-CoV-2-specific IgG4 levels after several COVID-19 mRNA vaccinations correlated with reduced Fc-mediated effector functions^6,7,12,33^, but these studies did not evaluate PLWH with higher risk of severe COVID-19.

In this study, we sought to determine whether PLWH may mount suboptimal antibody responses to sequential SARS-CoV-2 vaccinations by comparing the properties and functions of SARS-CoV-2-specific serum antibodies in elderly cohorts of PLWH on ART vs. PWOH (all male, median age of 68 and 66 years old, respectively) within one year after receiving the third dose of COVID-19 mRNA vaccine. HIV-1-specific antibodies in the PLWH group were evaluated for comparison. Using multiplex bead binding antibody assays, we measured the levels of antigen-specific total Ig antibody and the distribution of Ig isotypes and IgG subtypes (IgG1-4, IgA1, IgA2 and IgM). The capacities to bind complement and Fcγ receptor and mediate ADCP and ADCC were also evaluated. In addition, serum neutralization capacities were tested against several variants of concerns. The data demonstrated higher levels of SARS-CoV-2-specific IgG4 antibodies in PLWH vs. PWOH that correlated to less potent Fc-mediated antibody functions.

## Methods

### Human specimens

Serum samples were collected from 37 PLWH who received COVID-19 mRNA vaccines at James J. Peters VA Medical Center under IRB#1639479 (Supplemental Table 1). CD4 counts, measured at time of recruitment, ranged from 75–1261/mm^3^ (median: 625/mm^3^), whereas plasma vRNA loads were undetectable (< 20 copies/mL) or very low (22–145 copies/mL) for more than five years. Specimens from 24 PWOH who received COVID-19 mRNA vaccines were collected as part of the PARIS Study (IRB#20-03374) or under the Viral Sample Collection Protocol (IRB#16–00791) (Supplemental Table 2). Specimens obtained after the third dose of mRNA vaccine with SARS-CoV-2 spike of the Wuhan strain were tested. Age, sex and time of sample collection post vaccination were comparable for PLWH and PWOH: 100% male in both groups, median age of 68 and 66, and median sampling time of 5 and 5.7 months, respectively. However, 95% of PLWH identified as Black or African American and/or Hispanic compared to 22% of PWOH (Supplemental Table 3).

All experimental methods and protocols involving human samples were approved by the James J. Peters VA Medical Center IRB and the Icahn School of Medicine at Mount Sinai IRB and carried out in accordance with the approved protocols. All participants provided informed consent and signed written consent forms prior to sample and data collection. All participants provided permission for sample banking and sharing. All samples were de-identified and heat-inactivated before use in the study.

### Recombinant proteins

SARS-CoV-2 spike and RBD proteins were produced as described previously^34,35^. S1 (amino acids 16–685), S2 (amino acids 686–1213), and nucleoprotein (amino acids 1-419) antigens were purchased from ProSci Inc, CA (#97 − 087, #97 − 079 and #11–184, respectively). All SARS-CoV-2 antigens were of the parental Wuhan-Hu-1 or WA1 strain. The following reagent was obtained through the NIH HIV Reagent Program, Division of AIDS, NIAID, NIH: Human Immunodeficiency Virus 1 (HIV-1) gp120 Recombinant Protein (M.CON-S delta11 gp120), ARP-12,576, contributed by Drs. Barton F. Haynes and Hua-Xin Liao. HIV-1 AE.A244 gp120 protein was kindly provided by Dr. Faruk Sinangil (Global Solutions for Infectious Diseases) and recombinant HIV-1 B.HXB2 p24 protein was purchased from Abcam (#ab43037).

### Multiplex bead Ab binding assay

Measurement of total Ig and Ig isotypes to SARS-CoV-2 antigen-coupled beads was performed as described^8–11^. The quantification was based on median fluorescent intensity (MFI) values at the designated sample dilutions. For total Ig responses, serum specimens were diluted 10-fold from 1:100 to 1:100,000, reacted with antigen-coated beads, and treated sequentially with biotinylated anti-human total Ig antibodies and PE-streptavidin. The isotyping assays were performed at a 1:200 dilution using human Ig isotype- or subtype-specific antibodies.

For the C1q assay, beads with antigen-antibody complexes from serially diluted serum were incubated with the C1q component from human serum (Sigma, #C1740) for one hour at room temperature, followed by an anti-C1q-PE antibody (Santa Cruz, #sc-53544 PE) for 30 min at room temperature.

For the FcγR assays, antigen-antibody complexes from serially diluted serum were incubated with His-tagged recombinant FcγRIIIa/CD16a protein (R&D Systems™, #4325FC050) for one hour at room temperature, followed by an anti-His-PE antibody (R&D Systems™, #IC050P) for 30 min at room temperature.

The MFI binding levels were plotted to obtain titration curves, and areas-under the curves (AUC) were calculated.

### Replication-competent spike-VSV neutralization assay

This assay used BHK-hACE2 target cells which were seeded at a density of 12,000 cells per well in flat-bottom 96-well plates (Corning Falcon, #353072) and incubated at 37 °C/5% CO_2_ overnight (~ 20 h). On the following day, replication-competent vesicular stomatitis virus (VSV) virions expressing SARS-CoV-2 spike and a green fluorescent protein (GFP) reporter^36^ were pre-incubated with 4-fold serially diluted serum (starting from 1:10 to 1:40,960) in DMEM with 10% FBS and 1% PenStrep for a minimum of 10 min at room temperature, and then added to the target cells. At 13 h post-infection, GFP counts were acquired by the Celigo imaging cytometer (Nexcelom Biosciences, version 4.1.3.0).

To calculate ID_50_, GFP counts from “no serum” conditions were set to 100%; GFP counts of each serum-treated condition were normalized to no serum control. Inhibition curves were generated using Prism 9.1.2 (225) (GraphPad Software) with the “log (inhibitor) vs. normalized response-variable slope” model.

### Antibody-dependent cellular phagocytosis (ADCP)

Assays to measure spike-specific ADCP were performed using a protocol reported previously^9,10^. Briefly, FluoSpheres carboxylate-modified yellow-green fluorescent microspheres (Thermo Fisher, #F8823) were coupled with SARS-CoV-2 spike or RBD proteins using the xMAP Antibody Coupling Kit (5 µg protein/~36.4 × 10^9^ beads, Luminex #40-50016). Spike-conjugated microspheres were incubated with serially diluted serum for two hours at 37 °C in the dark. After washing and centrifugation (2,000 g, 10 min), the beads (~ 3 × 10^8^ beads, 10 µL/well) were incubated with THP-1 cells (0.25 × 10^5^ cells, 200 µL/well) for 16 h. The samples were analyzed on an Attune NxT flow cytometer (Thermo Fisher, #A24858). Data analysis was performed using FCS Express 7 Research Edition (De Novo Software). ADCP scores were calculated as follows: $\:\left(\mathrm{\%}\:\mathrm{m}\mathrm{i}\mathrm{c}\mathrm{r}\mathrm{o}\mathrm{s}\mathrm{p}\mathrm{h}\mathrm{e}\mathrm{r}\mathrm{e}\:\mathrm{p}\mathrm{o}\mathrm{s}\mathrm{i}\mathrm{t}\mathrm{i}\mathrm{v}\mathrm{e}\:\mathrm{c}\mathrm{e}\mathrm{l}\mathrm{l}\mathrm{s}\right)\:\mathrm{x}\:\left(\mathrm{g}\mathrm{e}\mathrm{o}\mathrm{m}\mathrm{e}\mathrm{t}\mathrm{r}\mathrm{i}\mathrm{c}\:\mathrm{m}\mathrm{e}\mathrm{a}\mathrm{n}\:\mathrm{f}\mathrm{l}\mathrm{u}\mathrm{o}\mathrm{r}\mathrm{e}\mathrm{s}\mathrm{c}\mathrm{e}\mathrm{n}\mathrm{t}\:\mathrm{i}\mathrm{n}\mathrm{t}\mathrm{e}\mathrm{n}\mathrm{s}\mathrm{i}\mathrm{t}\mathrm{y}\:\mathrm{o}\mathrm{f}\:\mathrm{t}\mathrm{h}\mathrm{e}\:\mathrm{m}\mathrm{i}\mathrm{c}\mathrm{r}\mathrm{o}\mathrm{s}\mathrm{p}\mathrm{h}\mathrm{e}\mathrm{r}\mathrm{e}\:\mathrm{p}\mathrm{o}\mathrm{s}\mathrm{i}\mathrm{t}\mathrm{i}\mathrm{v}\mathrm{e}\:\mathrm{c}\mathrm{e}\mathrm{l}\mathrm{l}\mathrm{s}\right)$/1000.

### Antibody-dependent cellular cytotoxicty (ADCC)

This assay used SARS-CoV-2 spike-expressing CHO-K1 cells (HaloTag^®^-HiBiT) (Promega, #CS3195A01) as target cells, which were seeded in F12 medium (Gibco, #11765) with 10% FBS (Seradigm, #89510) at a density of 2,500 cells per well in white, U-bottom, 96-well assay plates (Costar, #3355) and incubated at 37 °C/5% CO_2_ overnight (~ 20 h). On the following day, serially diluted serum in RPMI 1640 medium (Corning, #10-041-CV) with 5% FBS, 1:1000 2-mercaptoethanol 55mM (Gibco, #21985) and 5 ng/mL IL-2 (Sigma, #I2644) and 125,000 per well of ADCC-qualified human PBMC (Promega, #CS3055A01) were added to the target cells. After 4 h, luminescence was measured using Nano-Glo^®^ HiBiT Extracellular assay detection (Promega, #N2421) and ADCC scores were calculated relative to digitonin (Sigma, #D141), used as maximum release control.

### Ethics statement

All participants signed written consent forms prior to sample and data collection. All participants provided permission for sample banking and sharing.

### Statistics

Statistical analyses were performed as designated in the figure legends using GraphPad Prism 10 (GraphPad Software, San Diego, CA). Correlation matrices, principal components analyses and radart charts were generated using R version 4.4.3 (The R Foundation for Statistical Computing) and corrplot, prcomp and radarchart packages, respectively.

### Role of the funding source

The funding source had no involvement in study design and in the collection, analysis, and interpretation of data.

## Results

### Higher levels of SARS-CoV-2-specific total antibodies in Sera of PLWH vs. PWOH after COVID-19 mRNA vaccinations

Serum specimens were collected at one time point for each of 37 PLWH and 24 PWOH volunteers after the third dose of COVID-19 mRNA vaccine expressing SARS-CoV-2 Wuhan spike (median 5 and 5.7 months post vaccination, from March 2022 to September 2023). Age, sex and time of sample collection post vaccination were similar between the PLWH and PWOH groups (Supplemental Table 3). Notably, PLWH and PWOH were 100% male with median age of 68 and 66, respectively. However, race/ethnicity differed: 81% of PLWH identified as Black or African American and 19% identified as Hispanic or Latino as compared to 13% and 4% respectively for PWOH.

**Fig. 1:**
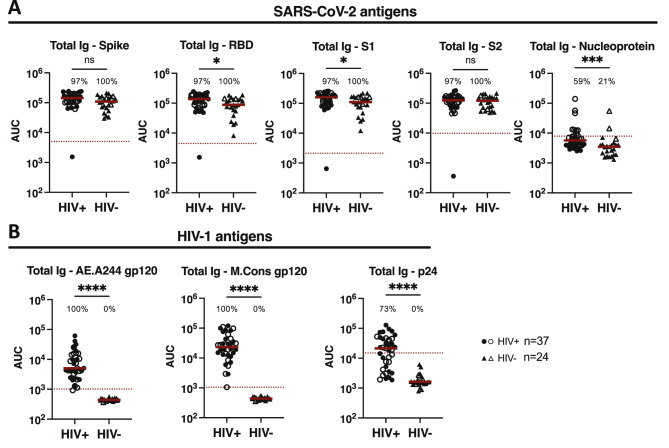
Total immunoglobulin (Ig) responses against SARS-CoV-2 (A) and HIV-1 (B) in sera of PLWH vs. PWOH after COVID-19 mRNA vaccinations. Total Ig responses against SARS-CoV-2 (spike, RBD, S1, S2, and nucleoprotein) (A) and HIV-1 (AE.A244 gp120, M.Cons gp120, and B.HXB2 p24) (B) antigens were measured in titrated sera from 37 PLWH (circles) vs. 24 PWOH (triangles). Sera were collected after three COVID-19 mRNA vaccinations (median: 5- and 5.7-months post vaccination for PLWH and PWOH, respectively). Pre-pandemic samples were tested as a negative control for the SARS-CoV-2 antigens while the PWOH samples were used as a negative control for the HIV-1 antigens. The percentages of responders above the cut-off values (based on negative control mean + 3SD) were indicated for each antigen. Dotted line: negative control mean + 3SD. Red line: median. AUC: area under the titration curve. ****, *p* < 0.0001; ***, *p* < 0.001; *, *p* < 0.05; ns *p* ≥ 0.05 by two-tailed Mann-Whitney test. Open symbols represent individuals with high-level anti-nucleoprotein total Ig.

Each specimen was titrated to measure total Ig antibody levels against SARS-CoV-2 Wuhan spike, RBD, S1, S2, and nucleoprotein antigens and HIV-1 gp120 (AE.A244 and M.Cons) and p24 (B.HXB2) antigens in a Luminex multiplex bead binding assay. Pre-pandemic samples served as a negative control for SARS-CoV-2 antigens, while PWOH samples were used as a negative control for HIV-1 antigens. Areas under the titration curves (AUC) for PLWH and PWOH were calculated and compared (Fig. 1). The data showed that PLWH generated higher levels of SARS-CoV-2-specific total Ig than PWOH, with significant differences detectable for RBD, S1 and nucleoprotein antigens (Fig. 1A). Sampling time points were comparable between the two groups, ranging from 1 to 12 months post 3rd vaccination (median: 5 months for PLWH and 5.7 months for PWOH, Supplemental Table 3). No significant correlation was observed between time post-vaccination and spike-specific Ig levels (*r* = -0.222, *p* > 0.05 for both PLWH and PWOH). As expected, HIV-1-specific total Ig levels were higher in PLWH vs. PWOH (Fig. 1B).

**Fig. 2:**
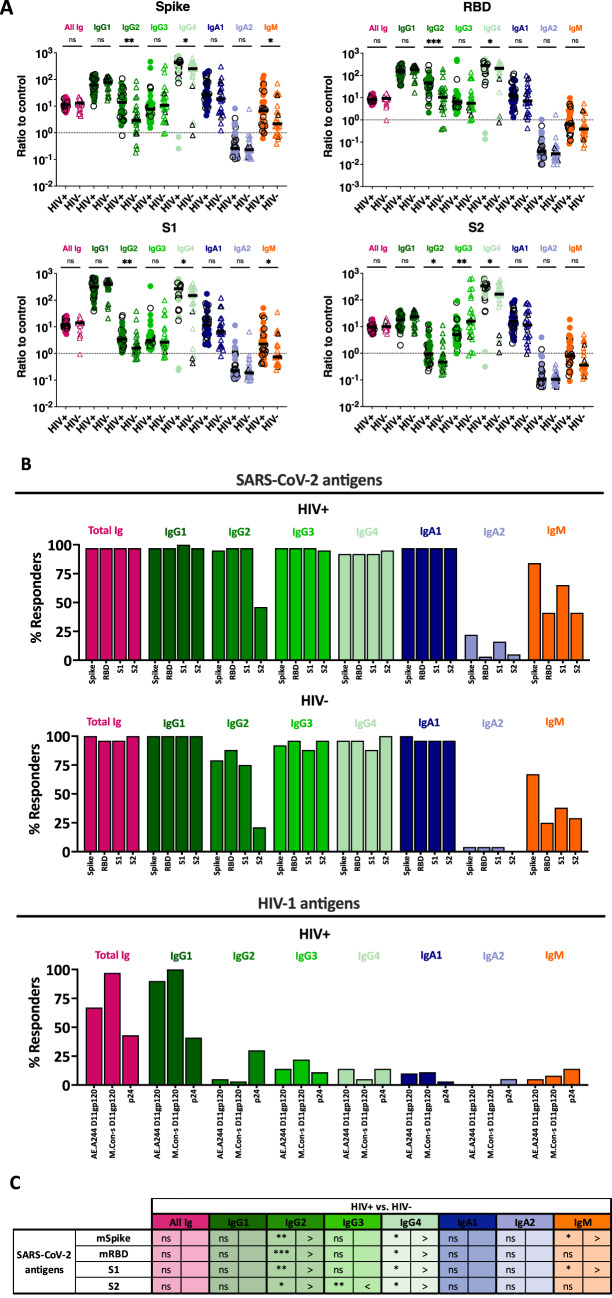
Antibody isotypes and subtypes against SARS-CoV-2 and HIV-1 antigens in sera of PLWH vs. PWOH after COVID-19 mRNA vaccinations. Total Ig, IgG1-4, IgA1, IgA2 and IgM against SARS-CoV-2 (spike, RBD, S1, S2, and nucleoprotein) and HIV-1 (gp120 and p24) antigens were evaluated in sera (1:200 dilution) from 36 PLWH and 24 PWOH. Pre-pandemic samples were tested as negative control for the SARS-CoV-2 antigens while the PWOH samples were used as negative control for the HIV-1 antigens. (**A**) Relative levels of total Ig, IgG1-4, IgA1, IgA2 and IgM against SARS-CoV-2 (spike, RBD, S1, S2, and nucleoprotein) and HIV-1 (gp120 and p24) antigens in sera of PLWH (circles) vs. PWOH (open triangles). Ratios of test samples over respective controls were used for comparison between the two groups. Ratios of 1 over control are marked by dotted lines. Black horizontal lines: median. Black open symbols represent individuals with high-level anti-nucleoprotein total Ig. (**B**) Percentages of responders who produced different antibody isotypes and subtypes against SARS-CoV-2 and HIV-1 antigens in sera of PLWH and PWOH after COVID-19 mRNA vaccinations. The cut-off values were determined based on mean + 3SD of negative controls. (**C**) Summary of antigen-specific Ig isotype/subtype profiles in PLWH vs. PWOH from data shown in panel (**B**). *** *p* < 0.001; ** *p* < 0.01; * *p* < 0.05; ns *p* ≥ 0.05 by Mann-Whitney test.

**Fig. 3:**
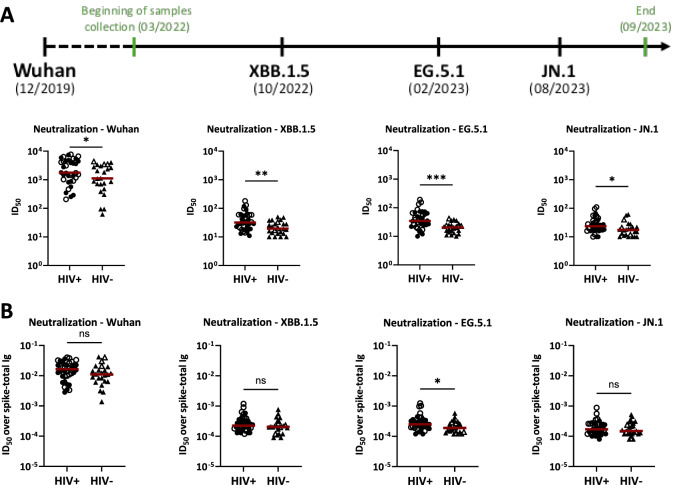
Neutralization levels in sera of PLWH vs. PWOH after COVID-19 mRNA vaccination. (**A**) Neutralization was measured using recombinant VSV expressing spike glycoproteins of Wuhan, XBB.1.5, EG.5.1 and JN.1 SARS-CoV-2 strains. The period of sample collection relative to the timeline of variant emergence is presented. Sera from 36 PLWH vs. 24 PWOH after three COVID-19 mRNA vaccinations were serially diluted to determine 50% inhibitory dilution (ID_50_) titers against each virus strain. (**B**) Neutralization potencies were determined by calculating the ratios of ID_50_ titers over spike-total Ig levels. Red lines denote median values. *** *p* < 0.001; ** *p* < 0.01; * *p* < 0.05 by Mann-Whitney test. Open symbols represent individuals with high-level anti-nucleoprotein total Ig.

**Fig. 4:**
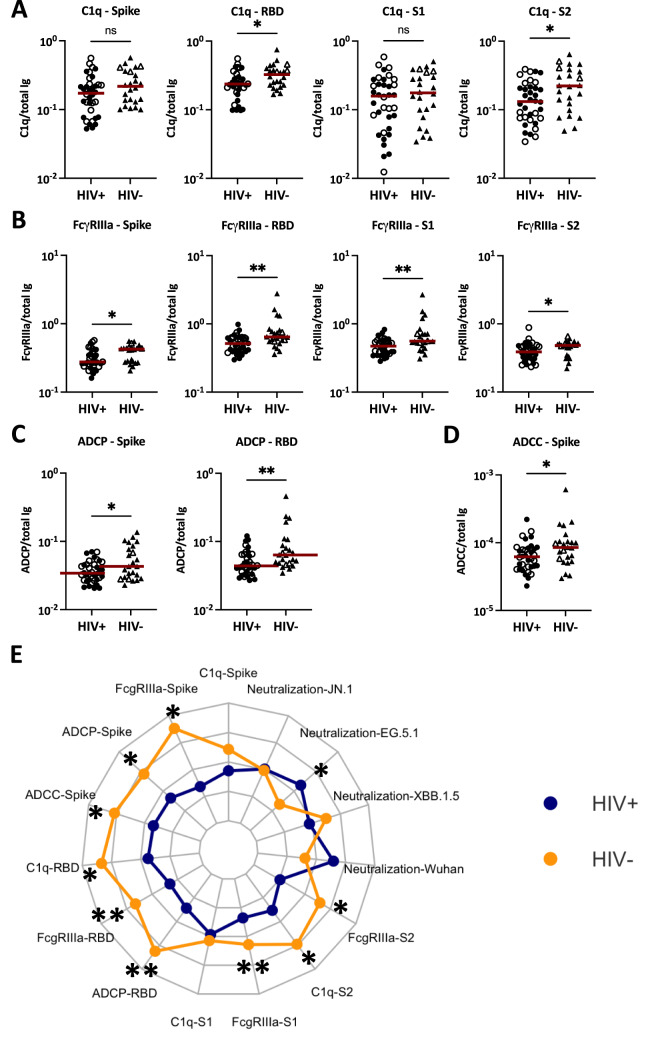
Fc-mediated activities of SARS-CoV-2-specific antibodies in sera of PLWH vs. PWOH after COVID-19 mRNA vaccinations. Fc functions of spike-, RBD, S1- and S2-specific antibodies were measured in serially diluted sera from 36 PLWH (circles) vs. 24 PWOH (triangles). Fc functional potencies were calculated by dividing Fc activity levels (AUC) with total Ig amounts (AUC) for the respective antigens. (**A**) C1q binding potencies of spike-, RBD, S1- and S2-specific antibodies. (**B**) FcγRIIIa binding potencies of spike-, RBD, S1- and S2-specific antibodies. (**C**) Spike- and RBD-specific ADCP activities. (**D**) Spike-specific ADCC activities. Red line: median. (**E**) Radar chart showing functional potencies of SARS-CoV-2-specific antibodies in 36 PWH (blue) vs. 24 PWOH (orange). Open symbols: individuals with high-level anti-nucleoprotein total Ig. ** *p* < 0.01; * *p* < 0.05; ns *p* ≥ 0.05 by Mann-Whitney test.

**Fig. 5:**
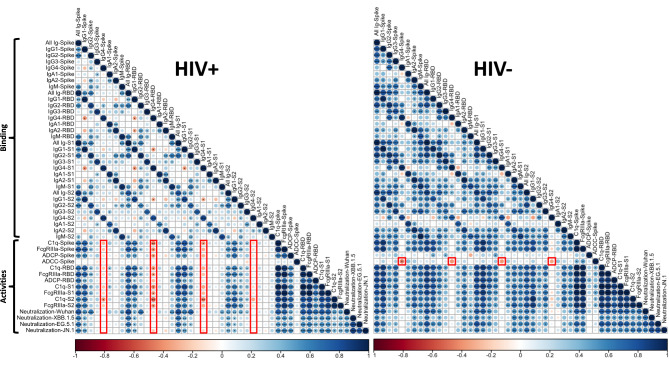
Correlations between SARS-CoV-2-specific binding and functional activities of sera antibodies in PLWH and PWOH groups after mRNA vaccinations. Spearman correlation matrices were generated to display the correlations of binding and functional activities with each other for PLWH (left) and PWOH (right) groups. The Spearman r values are indicated by color from red (negative correlation) to blue (positive correlation) and circle size (larger circles for larger positive or negative r values). **** *p* < 0.0001; *** *p* < 0.001; ** *p* < 0.01; * *p* < 0.05; no asterisk *p* ≥ 0.05.

When compared to the pre-pandemic controls, spike-, RBD-, S1- and S2- specific Ig responses were detectable in all PLWH and PWOH volunteers, except for one PLWH (Fig. 1A). All PLWH were also positive for gp120-specific total Ig, while 73% showed p24-specific total Ig at levels above controls (Fig. 1B). The PLWH volunteer who failed to mount antibody responses to SARS-CoV-2 spike upon vaccinations was able to maintain the responses to HIV-1 gp120 and p24 antigens. Despite ART-mediated HIV-1 control (viral load < 20 copies/mL), CD4 count remained low (75/mm^3^) and recurrent episodes of cryptococcal meningitis were reported. This individual also presented with multiple episodes of SARS-CoV-2 infection (confirmed with a Rapid COVID-19 Antigen Test at James J. Peters VA Medical Center) but did not generate antibody responses to SARS-CoV-2 nucleoprotein. This participant was therefore excluded from the subsequent analyses (Figs. 2, 3, 4 and 5).

In addition, it should be noted that 59% of PLWH and 21% of PWOH displayed nucleoprotein-specific total Ig responses (Fig. 1A), most likely due to pre-vaccination or breakthrough SARS-CoV-2 infections, although the numbers of reported or confirmed cases were only 16% and 17% among PLWH and PWOH, respectively, and the presence of anti-nucleoprotein antibodies did not align with infection history (Supplemental Tables 1 and 2). Throughout the subsequent experiments, this factor was taken into account by analyzing data from all individuals as well as data that exclude individuals with positive anti-nucleoprotein antibodies, and both analyses yielded compable results (Supplemental Fig. 1).

### Higher SARS-CoV-2-specific IgG2 and IgG4 levels in sera of PLWH vs. PWOH after COVID-19 mRNA vaccinations

Ig isotypes (IgG1-4, IgA1-2 and IgM) elicited against SARS-CoV-2 and HIV-1 antigens were then evaluated in the sera of PLWH vs. PWOH. Total Ig responses were tested in parallel as positive controls. In comparison to the pre-pandemic controls, spike-specific IgG1-4 and IgA1, but not IgA2, were detected in the vast majority of post-vaccination sera from both PLWH and PWOH, whereas IgM were present only in a proportion of PLWH and PWOH (Fig. 2A, B). Similar patterns were observed for RBD, S1, and S2 antigens. In comparison, IgG1 is the predominant IgG subtype reactive with gp120 and p24, while the other IgG subtypes and Ig isotypes were low or undetectable above control (Fig. 2B).

To compare the levels of spike-specific Ig isotypes and subtypes between PLWH and PWOH, the ratios over pre-pandemic control were calculated (Fig. 2A, C). The data showed that PLWH generated higher levels of SARS-CoV-2-specific IgG2 and IgG4 than PWOH, with significant differences detectable for spike, RBD, S1, and S2 antigens (Fig. 2A, C). In the case of S2 antigen, lower IgG3 levels were also observed in PLWH compared to PWOH. In addition, spike- and S1-specific IgM levels were higher in PLWH vs. PWOH. The levels of Ig isotypes and subtypes did not correlate with CD4 counts in PLWH (*p* > 0.05 for all tested Ig parameters). These data indicate a skewing IgG responses toward spike-specific IgG2 and IgG4 with poor Fc activities and away from IgG3 with high Fc potencies in PLWH.

### Comparable SARS-CoV-2 virus neutralization activities in Sera of PLWH vs. PWOH after COVID-19 mRNA vaccinations

The neutralization potencies of antibodies in sera of PLWH vs. PWOH after mRNA vaccinations were assessed using recombinant VSV viruses expressing spike proteins of Wuhan (WT) and three variants of concern that circulated during the period of sample collection (XBB.1.5, EG.5.1, JN.1). Serum neutralization ID_50_ titers against all four strains were higher in PLWH vs. PWOH (Fig. 3A). However, since PLWH generated higher levels of SARS-CoV-2-specific antibodies (Fig. 1A), we normalized the neutralization ID50 titers to the antibody levels and observed that the antibodies induced in PLWH and PWOH displayed similar potencies to neutralize Wuhan, XBB1.5, and JN.1 (Fig. 3B). A slightly greater capacity to neutralize EG.5.1 was still apparent in PLWH compared to PWOH, but this difference was not detected when individuals with high-level anti-nucleoprotein were excluded (Supplemental Fig. 1).

### Lower Fc-mediated activities of SARS-CoV-2-specific serum antibodies from PLWH vs. PWOH after COVID-19 mRNA vaccinations

The potencies of Fc-mediated activities mediated by SARS-CoV-2-specific serum antibodies in the PLWH and PWOH groups after COVID-19 mRNA vaccinations were subsequently evaluated by measuring C1q binding, FcγR binding, ADCP and ADCC (Fig. 4). To account for variable antigen-specific antibody levels in individual samples from both groups, the ratios of Fc activities over the level of total Ig against the respective antigens were calculated. A trend towards lower SARS-CoV-2-specific C1q binding activities was observed in the PLWH vs. PWOH group, with significiant differences observed for RBD and S2 antigens (Fig. 4A). More striking differences were noted for FcγR binding activities such that FcγRIIIa binding potencies was significantly lower in PLWH vs. PWOH for antibodies against all four spike antigens tested (Fig. 4B). In addition, the ADCP data demonstrated lower Fc-dependent functional potencies of both spike- and RBD-specific antibodies in PLWH vs. PWOH (Fig. 4C). Finally, ADCC activities of spike-specific antibodies were also lower in PLWH vs. PWOH (Fig. 4D). The reduced Fc activities of SARS-CoV-2-specific antibodies in PLWH vs. PWOH remained evident after excluding those with high levels of anti-nucleoprotein antibodies (Supplemental Fig. 1). Of note, no correlation was observed between Fc potencies and CD4 counts in PLWH (*p* > 0.05 for all parameters tested).

Altogether, a radar plot consolidating data of antibody functions in Figs. 3 and 4 provided a graphical demonstration that while virus-neutralizing activities were similar in PLWH and PWOH, the Fc functional potencies were compromised in the PLWH group (Fig. 4E).

### Correlation and principal component analyses of SARS-CoV-2-specific binding and functional antibody activities to distinguish PLWH from PWOH

Correlation matrices for all tested SARS-CoV-2-specific antibody binding levels and functional capacities further revealed distinct features between the PLWH and PWOH groups (Fig. 5).  A reduction in the number and strength of positive correlations was observed in PLWH vs. PWOH (Fig. 5). Notably, RBD-specific IgG4 levels showed strong negative correlations with Fc-dependent activities in the PLWH group (Fig. 5), in line with inherently poor Fc functionality of IgG4. RBD-specific IgG4 levels also correlated negatively with ADCC potencies in the PWOH group (Fig. 5). On the other hand, IgG1 levels against spike, RBD, S1, and S2 antigens positively correlated with Fc functions and neutralization in both groups.

> and < denote higher and lower levels in PLWH vs. PWOH.

Lastly, principal component analyses on binding (Supplemental Fig. 2A) and functional activities (Supplemental Fig. 2B) of antibodies from PLWH and PWOH also demonstrated that SARS-CoV-2-specific functional potencies, and not binding levels, were able to clearly differentiate between the PLWH and PWOH groups.

## Discussion

By comparing the properties and functions of SARS-CoV-2-specific serum antibodies in elderly male cohorts of PLWH on ART and PWOH within one year after receiving the third dose of COVID-19 mRNA vaccine, we demonstrated that, despite higher levels of SARS-CoV-2-specific total Ig in PLWH, their Fc potencies were lower than those of PWOH. In contrast, neutralization potencies were similar between the two groups. Notably, lower Fc potencies in PLWH were specifically correlated with higher levels of SARS-CoV-2-specific IgG4 and were not associated with lower CD4 counts in this group (median CD4 count: 625/mm^3^). Fc functionalities were indeed the distinguishing features between PLWH and PWOH, driven by differences in IgG subtypes, especially IgG4. Of note, elevated IgG4 responses in PLWH were directed against SARS-CoV-2 spike after mRNA vaccination, while IgG4 responses to HIV-1 antigens were negligible. The basis for heightened spike-specific IgG4 responses in PLWH vs. PWOH is yet unknown. Chronic immune activation, a hallmark of HIV-1 infection, may predispose PLWH to intensified IgG4 responses after repeated mRNA vaccination. IgG4, typically regarded as a marker of Th2 responses, has been linked to prolonged, repeated, or high-dose antigen exposure, as observed in allergies, autoimmune diseases, and parasitic infections^37,38^. These findings underscore the importance of defining IgG subclass distribution and skewing in evaluating vaccine-induced responses in PLWH.

The role of IgG4 responses to vaccines against infectious pathogens has not been much studied. In the field of HIV-1 vaccine research, repeated immunizations as performed in the VAX003 trial of a recombinant bivalent gp120 protein (AIDSVAX^®^ B/E) vaccine were shown to generate higher levels of HIV-1 gp120-specific IgG2 and IgG4 antibodies after seven vaccine doses^39,40^. In contrast, a prime-boost immunization with a canarypox vector (ALVAC-HIV) and the same AIDSVAX B/E vaccine in the RV144 trial^41^ resulted in higher HIV-specific IgG3 responses that correlated with reduced risk of HIV-1 acquisition and enhanced Fc effector functions such as ADCC and ADCP^40,42^. On the other hand, vaccine-induced IgG4 affected these effector functions negatively^40^. The induction of virus-specific IgG4 responses has also been shown upon repeated Pfizer or Moderna COVID-19 mRNA vaccination in healthy adults^7–11,13–15^ and after repeated administration of vaccines against pertussis and malaria^6^. Herein we demonstrated an increase in SARS-CoV-2-specific IgG4 and also IgG2 responses in elderly cohorts of PLWH vs. PWOH after three COVID-19 mRNA vaccinations. Nonetheless, the mechanisms leading to the augmented responses of poorly Fc functional IgG responses are not yet understood. Analyses of HIV-specific antibodies in the same individuals revealed predominance of IgG1 without elevated IgG4 and IgG2 responses. A study after vaccination with tetanus toxoid-conjugated capsular polysaccharides of Haemophilus influenzae type b demonstrated lower vaccine-specific IgM and IgG1 in PLWH with low CD4 counts (< 100/mm^3^) than in PWOH but the vaccine-specific IgG2 levels were similar, and no difference was seen when comparing PLWH with CD4 counts > 100/mm^3^ vs. PWOH^43^. A more recent study on the 2009 swine influenza A (H1N1) monovalent vaccine demonstrated that PLWH were more likely to have IgG2 deficiency than PWOH but did not correlate with poor responses to the vaccine^44^. The influenza-specific antibodies were mostly of IgG1 subtype for both groups and levels of IgG2, IgG3 and IgG4 were similarly low. In addition, no association was found between the pre-vaccination levels of total or influenza-specific IgG subtype levels and the antibody response generated by H1N1 vaccination in either group.

With respect to the responses to viral infections, virus-specific IgG4 antibodies are not prominently induced. In response to respiratory syncytial virus (RSV), virus-specific IgG4 is minimally elicited during acute infections and after repeated infections^13^. Chronic human cytomegalovirus infections also do not trigger significant IgG4 antibodies^45^. However, measles-specific IgG4 antibodies have been detected following natural infection^46^. A few reports also showed the induction of IgG4 after SARS-CoV-2 infection, although the prevailing antibody subtypes were IgG1 and IgG3 ^8^. Data from a Brazilian cohort during the early phase of the COVID-19 pandemic correlated an early onset and high levels of spike-specific IgG4 antibodies with a more severe disease progression after SARS-CoV-2 infection, implicating the IgG4 contribution to less effective antiviral responses^49^. Another study reported a significant association of high IgG4/IgG1 ratios with poor disease outcome^50^. However, the causality cannot be addressed from correlative data as it is possible that a more severe infection leads to an IgG4 response or vice versa.

In our study we observed a significant inverse correlation between higher levels of SARS-CoV-2-specific IgG4 antibodies and lowered Fc-mediated functional potencies as measured by multiple Fc-dependent functions. These data are in line with the known poor Fc effector functions of IgG4 ^37^. However, the relative antiviral efficacy of IgG4 compared to the other IgG subtypes remains unclear and warrants further investigation. The assays employed in this study did not measure Fc-dependent activities against infectious virions or virus-infected cells, thereby limiting direct evaluation of antiviral Fc effector functions. The implications in protection against infection or disease also remain uncertain. Notably, a recent longitudinal study of primary healthcare workers in the CovidCatCentral cohort (Barcelona, Spain) reported that higher post-vaccination ratios of IgG4 + IgG2 over IgG1 + IgG3 correlated with increased risk of symptomatic COVID-19, whereas neutralization activity and FcγR binding were associated with a reduced infection risk^52^.

The study also has additional limitations. It was cross-sectional in design, involved small sample sizes, lacked longitudinal follow-up, precluding assessment of IgG4 durability over time or vaccine efficacy against repeated SARS-CoV-2 infection and severe COVID-19. The two groups were matched for age (median of 68 and 66) and sex (all male), but they differed in vaccine type (100% Moderna for PLWH and 83% Pfizer for PWOH) and in race/ethnic compositions (95% Black or African American and/or Hispanic among PLWH and 72% White among PWOH), raising the possibility that these factors contributed to differences in IgG subtype profiles and the observed enhanced IgG4 responses. Indeed, data from a centralized clinical data warehouse (Partners Research Patient Data Registry) revealed higher levels of total IgG, IgG1 and IgG3 in Asian and Black vs. White patients, while IgG2 and IgG4 levels were higher for Asians but comparable between Black and White patients^53^. While these data reflect steady-state levels, rather than vaccine-induced or antigen-specific or antibodies, they suggest that baseline IgG4 differences between Black and White individuals are unlikely to account for the difference observed in our study. Moreover, this study examined samples only from male volunteers, limiting the generalizability of the findings to female PWLH and PWOH.

Vaccine type may also influence IgG subtype responses and functions. A randomized controlled trial in PLWH reported similar antibody titers, neutralization activities and vaccine efficacy between Moderna vs. Pfizer vaccine recipients, but IgG subtypes and Fc functions were not evaluated^54^. Another study involving 245 vaccinated individuals found similar levels of FcγR binding antibodies and Fc functions (ADCP and ADNP) after two doses of Moderna vs. Pfizer vaccines^55^, but only data after the second dose, not the third dose as in our study, were reported. More recent studies indicate that elevated SARS-CoV-2-specific IgG2 and IgG4 responses were more pronounced in young adults after a third dose of the Pfizer vs. Moderna vaccines^15^. In contrast, another report found that elderly Moderna vaccine recipients produced higher IgG4 and, to a lesser extent, IgG2 levels and showed lower ADCC compared to Pfizer vaccine recipients^56^, suggesting that both vaccine type and age may influence IgG subclass switching and Fc effector capacity. Further studies are needed to characterize the Fc profiles and functional potencies of vaccine-induced antibodies in PLWH, across different age groups, after repeated doses of Moderna vs. Pfizer vaccines. The data may clarify whether one vaccine offers superior Fc-dependent immunity for PLWH or whether further vaccine optimization, including alternative vaccine platforms such as protein or virus-vectored vaccines, is warranted. Lastly, alterations in Fc glycosylation, especially fucosylation, galactosylation, and sialyation, are known to modulate FcγR- and complement-mediated effector functions^57^. The glycan alterations may evolve with repeated mRNA vaccination and shape antibody functionality, but they were not evaluated in this study^7,57,58^.

In summary, our study demonstrated that, following three COVID-19 mRNA vaccinations, elderly male PLWH as compared to PWOH mounted lower Fc-dependent antibody responses that were associated with increased levels of SARS-CoV-2-specific IgG4. Fc functionalities have been implicated in immune protection against many viral diseases^55,59–62^; however, the extent to which reduced Fc effector potencies impact COVID-19 mRNA vaccine effectiveness is unknown and requires additional investigation. Further research is also warranted to understand the mechanisms underlying differential IgG class switches in the context of mRNA vs. other vaccine platforms.

## Supplementary Information

Below is the link to the electronic supplementary material.

Supplementary Material 1

## Data Availability

Data is provided within the manuscript or supplementary information files.
