# Correction: Reduced Fc-mediated antibody responses after COVID-19 mRNA vaccination in a cohort of people living with HIV-1

**DOI:** 10.1038/s41598-025-33524-3

**Published:** 2026-02-03

**Authors:** Jéromine Klingler, Priyanka Gadam Rao, Juan C. Bandres, Ismael Pena, Katherine Bolanos Roldan, Gagandeep Singh, Brian Monahan, Charles Gleason, Yuexing Chen, Stefan Slamanig, Weina Sun, Chitra Upadhyay, Catarina E. Hioe

**Affiliations:** 1 James J. Peters VA Medical Center, Bronx, NY USA; 2 Division of Infectious Diseases, Department of Medicine, Icahn School of Medicine at Mount Sinai, New York, NY USA; 3 Department of Microbiology, Icahn School of Medicine at Mount Sinai, New York, NY USA; 4 Center for Vaccine Research and Pandemic Preparedness, Icahn School of Medicine at Mount Sinai, New York, NY USA

Correction to: *Scientific Reports* 10.1038/s41598-025-26149-z, published online 25 November 2025

The original version of this Article contained an error in Figures 2–4 where rendering caused blurring of the images.

The original Figures 2, 3 and 4 and accompanying legends appear below.

**Fig. 2:**
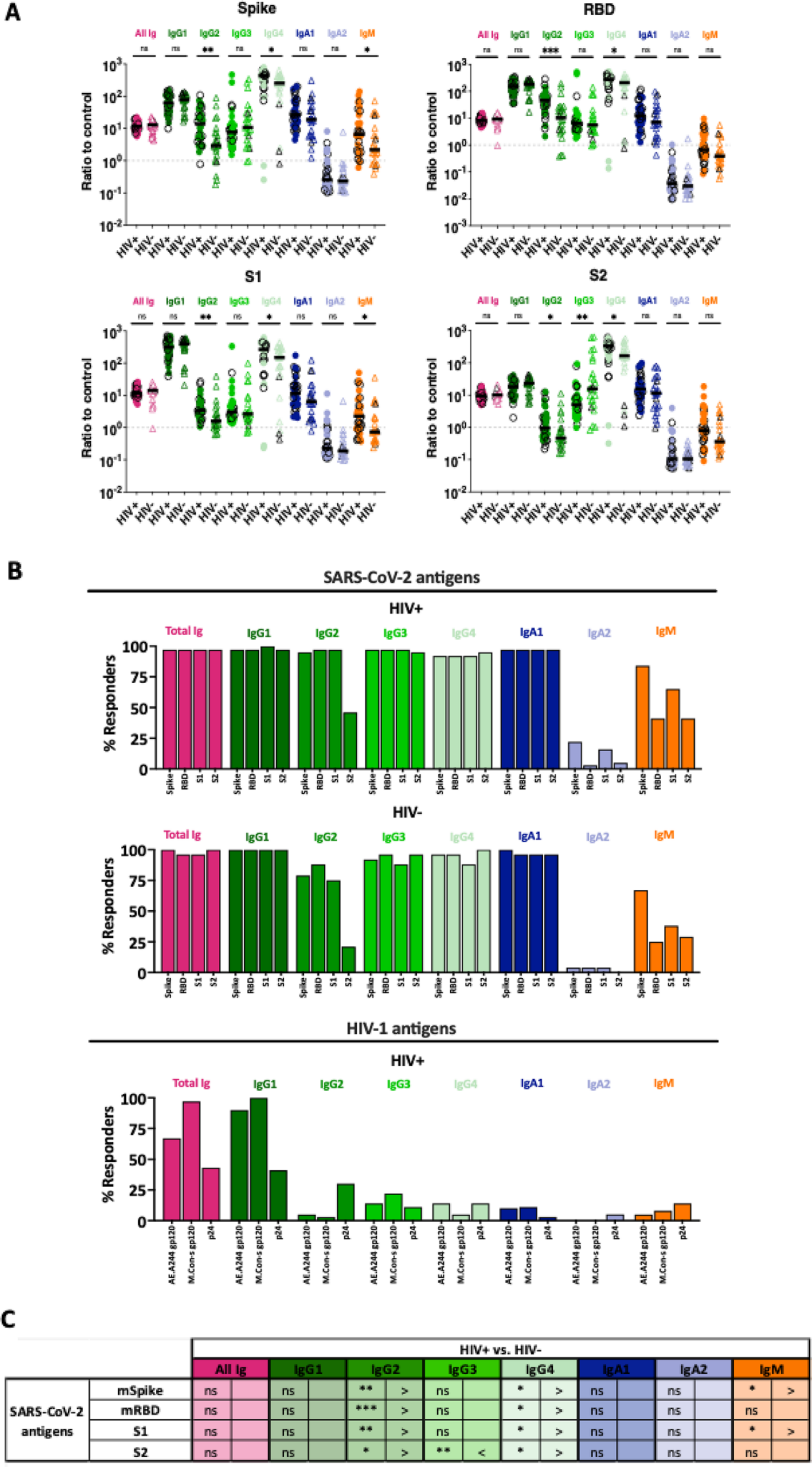
Antibody isotypes and subtypes against SARS-CoV-2 and HIV-1 antigens in sera of PLWH vs. PWOH after COVID-19 mRNA vaccinations. Total Ig, IgG1-4, IgA1, IgA2 and IgM against SARS-CoV-2 (spike, RBD, S1, S2, and nucleoprotein) and HIV-1 (gp120 and p24) antigens were evaluated in sera (1:200 dilution) from 36 PLWH and 24 PWOH. Pre-pandemic samples were tested as negative control for the SARS-CoV-2 antigens while the PWOH samples were used as negative control for the HIV-1 antigens. (**A**) Relative levels of total Ig, IgG1-4, IgA1, IgA2 and IgM against SARS-CoV-2 (spike, RBD, S1, S2, and nucleoprotein) and HIV-1 (gp120 and p24) antigens in sera of PLWH (circles) vs. PWOH (open triangles). Ratios of test samples over respective controls were used for comparison between the two groups. Ratios of 1 over control are marked by dotted lines. Black horizontal lines: median. Black open symbols represent individuals with high-level anti-nucleoprotein total Ig. (**B**) Percentages of responders who produced different antibody isotypes and subtypes against SARS-CoV-2 and HIV-1 antigens in sera of PLWH and PWOH after COVID-19 mRNA vaccinations. The cut-off values were determined based on mean + 3SD of negative controls. (**C**) Summary of antigen-specific Ig isotype/subtype profiles in PLWH vs. PWOH from data shown in panel (**B**). ***p < 0.001; **p < 0.01; *p < 0.05; ns *p* ≥ 0.05 by Mann–Whitney test.

**Fig. 3:**
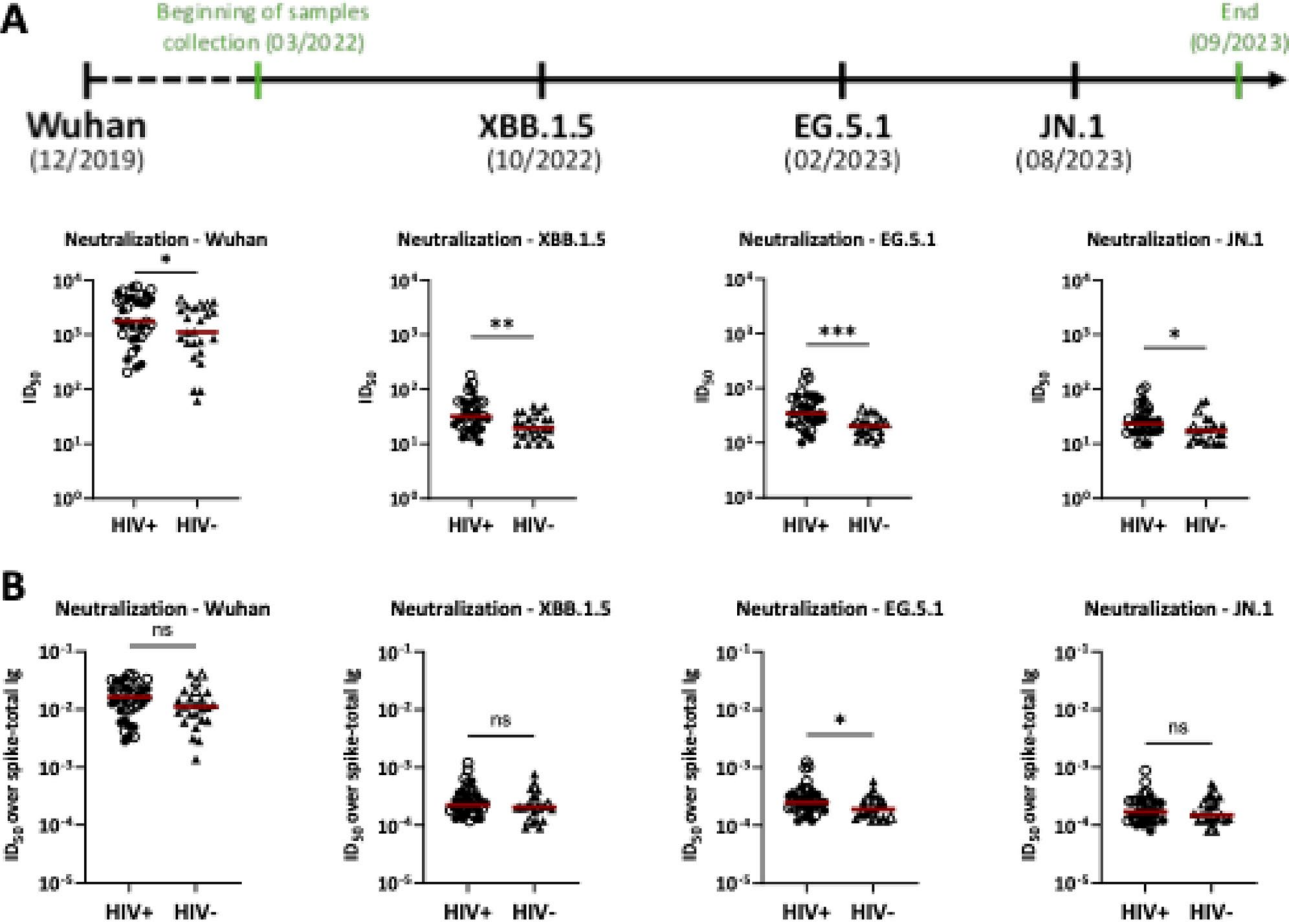
Neutralization levels in sera of PLWH vs. PWOH after COVID-19 mRNA vaccination. (**A**) Neutralization was measured using recombinant VSV expressing spike glycoproteins of Wuhan, XBB.1.5, EG.5.1 and JN.1 SARS-CoV-2 strains. The period of sample collection relative to the timeline of variant emergence is presented. Sera from 36 PLWH vs. 24 PWOH after three COVID-19 mRNA vaccinations were serially diluted to determine 50% inhibitory dilution (ID50) titers against each virus strain. (**B**) Neutralization potencies were determined by calculating the ratios of ID50 titers over spike-total Ig levels. Red lines denote median values. ***p < 0.001; **p < 0.01; *p < 0.05 by Mann–Whitney test. Open symbols represent individuals with high-level anti-nucleoprotein total Ig.

**Fig. 4:**
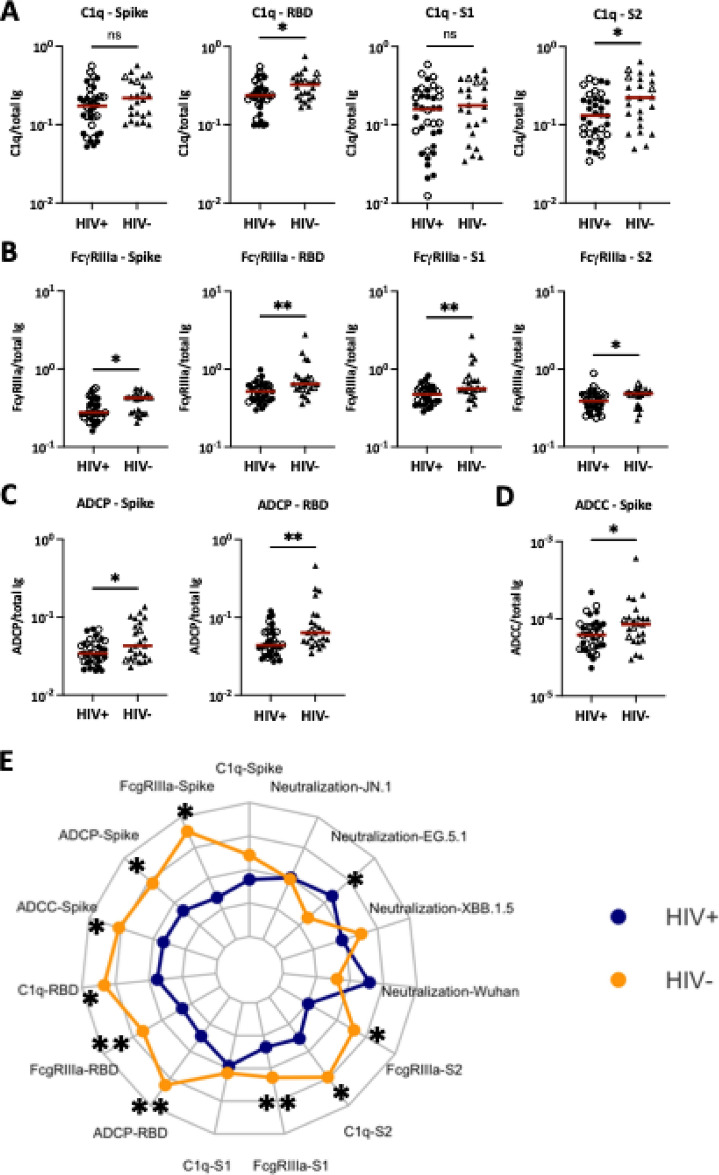
Fc-mediated activities of SARS-CoV-2-specific antibodies in sera of PLWH vs. PWOH after COVID-19 mRNA vaccinations. Fc functions of spike-, RBD, S1- and S2-specific antibodies were measured in serially diluted sera from 36 PLWH (circles) vs. 24 PWOH (triangles). Fc functional potencies were calculated by dividing Fc activity levels (AUC) with total Ig amounts (AUC) for the respective antigens. (**A**) C1q binding potencies of spike-, RBD, S1- and S2-specific antibodies. (**B**) FcγRIIIa binding potencies of spike-, RBD, S1- and S2-specific antibodies. (**C**) Spike- and RBD-specific ADCP activities. (**D**) Spike-specific ADCC activities. Red line: median. (**E**) Radar chart showing functional potencies of SARS-CoV-2-specific antibodies in 36 PWH (blue) vs. 24 PWOH (orange). Open symbols: individuals with high-level anti-nucleoprotein total Ig. **p < 0.01; *p < 0.05; ns *p* ≥ 0.05 by Mann–Whitney test.

The original Article has been corrected.

