# Supplementary material for: Reduced Fc-mediated antibody responses after COVID-19 mRNA vaccination in a cohort of people living with HIV-1
Source: Sci Rep. 2025 Nov 25;15:41988. doi: 10.1038/s41598-025-26149-z (PMC12647704; doi:10.1038/s41598-025-26149-z)
Supplement: Supplementary file 1 — Supplementary Material 1 [file 41598_2025_26149_MOESM1_ESM.pdf]

Suppl Figure 1

HIV+ vs. HIV-

Isotyping	All Ig				IgG1				IgG2				IgG3			
Antigen	All		Low anti-nucleoprotein		All		Low anti-nucleoprotein		All		Low anti-nucleoprotein		All		Low anti-nucleoprotein	
Spike	ns		ns		ns		ns		**	>	**	>	ns		ns	
RBD	ns		ns		ns		ns		***	>	**	>	ns		ns	
S1	ns		ns		ns		ns		**	>	**	>	ns		ns	
S2	ns		ns		ns		ns		*	>	*	>	**	<	*	<

Isotyping	IgG4				IgA1				IgA2				IgM			
Antigen	All		Low anti-nucleoprotein		All		Low anti-nucleoprotein		All		Low anti-nucleoprotein		All		Low anti-nucleoprotein	
Spike	*	>	*	>	ns		ns		ns		ns		*	>	*	>
RBD	*	>	*	>	ns		ns		ns		ns		ns		*	>
S1	*	>	*	>	ns		ns		ns		ns		*	>	*	>
S2	*	>	ns		ns		ns		ns		ns		ns		ns	

Neutralization				
Virus	All		Low anti-nucleoprotein	
Wuhan	ns		ns	
XBB.1.5	ns		ns	
EG.5.1	*	>	ns	
JN.1	ns		ns	

C1q				
Antigen	All		Low anti-nucleoprotein	
Spike	ns		ns	
RBD	*	<	*	<
S1	ns		ns	
S2	*	<	ns	

FcγRIIIa				
Antigen	All		Low anti-nucleoprotein	
Spike	*	<	**	<
RBD	**	<	**	<
S1	**	<	**	<
S2	*	<	*	<

ADCP				
Antigen	All		Low anti-nucleoprotein	
Spike	*	<	*	<
RBD	**	<	**	<

ADCC				
Antigen	All		Low anti-nucleoprotein	
Spike	*	<	ns	

Suppl Fig 1. Comparison of antigen-specific immunoglobulin types/subtypes and antibody activities in PWH vs. PWOH with or without individuals with high-level anti-nucleoprotein total Ig.

*** p <0.001; ** p <0.01; * p <0.05; ns p ≥0.05 for comparison between PHW and PWOH by Mann-Whitney test.

> and < denote significantly higher and lower levels in PWH vs. PWOH.

Suppl Figure 2

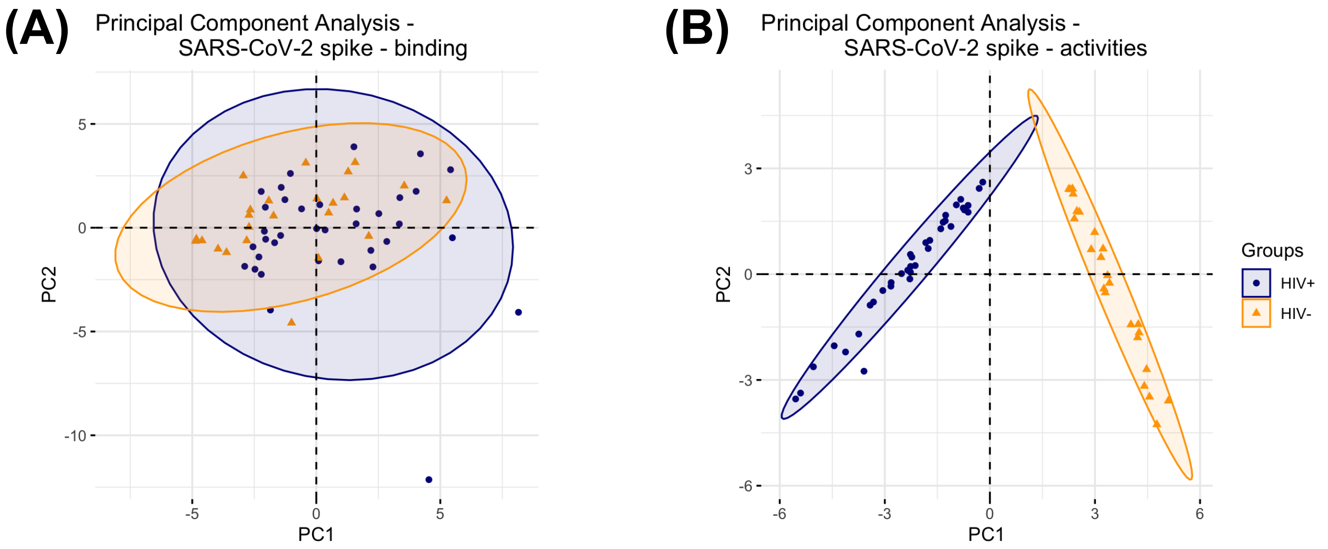

Suppl Fig 2. Principal component analyses (PCA) of antibody binding and functional potencies against SARS-CoV-2 spike in PWH vs. PWOH after COVID-19 mRNA vaccinations.
PCA were performed to compare all antibody parameters tested in this study. PCA data for antigen-binding levels (A) and functional potencies (B) of SARS-COV-2-spike specific sera antibodies are presented for 36 PWH (blue circles) vs. 24 PWOH (orange triangles) who received three COVID-19 mRNA vaccinations.

Supplemental Table 1: Demographic, clinical, and vaccinaiton data for PWH (n = 37)

Code	Age	Sex at birth	Race	Ethnicity	CD4 count (cells/mm^3)	Viral load (copies/mL)	Other clinical info	COVID-19 mRNA Vaccine Dose 3	Months to COVID-19 Vaccine Dose 3	SARS-CoV-2 infection	Nucleoprotein-specific total Ig levels above background
**JB001**	70-74	Male	Black or African American	Not Hispanic or Latino	900	<20	HCV	Moderna	4	None	Yes
**JB003**	80-84	Male	Black or African American	Not Hispanic or Latino	967	<20		Moderna	4	None	No
**JB004**	55-59	Male	Black or African American	Not Hispanic or Latino	1200	<20		Moderna	4	None	Yes
**JB005**	75-79	Male	White	Hispanic or Latino	1000	<20		Moderna	3	None	No
**JB006**	75-79	Male	White	Not Hispanic or Latino	676	<20	Chronic kidney disease (CKD)	Moderna	4	None	No
**JB007**	75-79	Male	White	Hispanic or Latino	575	<20	Coronary artery disease (CAD)	Moderna	7	None	No
**JB008**	55-59	Male	Black or African American	Not Hispanic or Latino	340	<20		Moderna	3	None	No
**JB009**	70-74	Male	Black or African American	Hispanic or Latino	307	<20		Moderna	4	None	Yes
**JB010**	70-74	Male	Black or African American	Not Hispanic or Latino	703	<20		Moderna	6	None	No
**JB011**	65-69	Male	White	Hispanic or Latino	764	<20		Moderna	2	None	No
**JB012**	45-9	Male	Black or African American	Not Hispanic or Latino	960	<20		Moderna	4	None	No
**JB013**	60-64	Male	Black or African American	Not Hispanic or Latino	716	<20	Lung cancer	Moderna	4	None	Yes
**JB014**	80-84	Male	Black or African American	Not Hispanic or Latino	795	<20	Chronic kidney disease (CKD)	Moderna	4	None	No
**JB015**	80-84	Male	Black or African American	Not Hispanic or Latino	625	<20		Moderna	5	None	No
**JB016**	65-69	Male	Black or African American	Not Hispanic or Latino	215	<20		Moderna	5	None	Yes
**JB017**	60-64	Male	Black or African American	Not Hispanic or Latino	814	<20		Moderna	4	None	Yes
**JB018**	70-74	Male	Black or African American	Not Hispanic or Latino	484	<20		Moderna	5	Post-dose 3	No
**JB019**	65-69	Male	Black or African American	Not Hispanic or Latino	400	<20	Adenocarcinoma	Moderna	5	None	No
**JB020**	70-74	Male	Black or African American	Not Hispanic or Latino	531	<20	Lymphoma	Moderna	5	Post-dose 3	No
**JB021**	60-64	Male	Black or African American	Not Hispanic or Latino	337	<20		Moderna	4	None	Yes
**JB022**	65-69	Male	Asian	Not Hispanic or Latino	525	102		Moderna	6	None	Yes
**JB023**	65-69	Male	African American	Not Hispanic or Latino	1232	<20		Moderna	4	None	Yes
**JB024**	70-74	Male	African American	Not Hispanic or Latino	379	<20	HBV	Moderna	5	None	Yes
**JB025**	65-69	Male	White	Hispanic or Latino	940	<20		Moderna	9	Post-dose 3	No
**JB026**	65-69	Male	Black or African American	Not Hispanic or Latino	396	<20	Cirrhosis	Moderna	7	None	No
**JB027**	70-74	Male	White	Hispanic or Latino	568	145		Moderna	8	None	No
**JB028**	50-54	Male	Black or African American	Not Hispanic or Latino	75	<20	Cryptococcal meningitis	Moderna	6	Post-dose 3	No
**JB029**	65-69	Male	Black or African American	Not Hispanic or Latino	195	76	HBV	Moderna	8	Post-dose 3	No
**JB030**	65-69	Male	Black or African American	Not Hispanic or Latino	743	<20		Moderna	7	Post-dose 3	No
**JB031**	65-69	Male	Black or African American	Not Hispanic or Latino	438	26	Prostate cancer	Moderna	7	None	No
**JB032**	40-44	Male	Black or African American	Not Hispanic or Latino	503	66		Moderna	7	None	Yes
**JB033**	65-69	Male	Black or African American	Hispanic or Latino	641	76		Moderna	9	None	No
**JB035**	75-79	Male	Black or African American	Not Hispanic or Latino	595	44	Lymphoma	Moderna	6	None	No
**JB036**	50-54	Male	Black or African American	Not Hispanic or Latino	659	<20		Moderna	9	None	Yes
**JB038**	60-64	Male	Black or African American	Not Hispanic or Latino	498	<20		Moderna	9	None	Yes
**JB041**	75-79	Male	Black or African American	Not Hispanic or Latino	1261	22		Moderna	12	None	Yes
**JB045**	60-64	Male	Black or African American	Not Hispanic or Latino	726	116	HBV	Moderna	12	None	Yes

Supplemental Table 2: Demographic and clinical data for PWOH (n = 24)

Code	Age	Sex at birth	Race	Ethnicity	COVID-19 mRNA Vaccine Dose 3	Months to COVID-19 Vaccine Dose 3	SARS-CoV-2 Infection	Nucleoprotein-specific total Ig levels above background
**61522**	55-59	Male	White	Hispanic or Latino	Moderna	1	Post-dose 3	No
**71485**	55-59	Male	Black or African American	Not Hispanic or Latino	Moderna	1	Post-dose 3	No
**37666**	50-54	Male	White	Not Hispanic or Latino	Pfizer	1	Post-dose 3	No
**58558**	60-64	Male	White	Not Hispanic or Latino	Pfizer	1	Post-dose 3	No
**70331**	50-54	Male	White	Not Hispanic or Latino	Pfizer	2	Post-dose 3	No
**10802**	50-54	Male	Black or African American	Not Hispanic or Latino	Pfizer	1	Post-dose 3	No
**75757**	60-64	Male	White	Not Hispanic or Latino	Pfizer	0	Unknown	No
**63967**	60-64	Male	White	Not Hispanic or Latino	Pfizer	1	Unknown	No
**86759**	50-54	Male	Other	Unknown	Pfizer	0	Unknown	No
**81369**	85-89	Male	Unknown	Unknown	Pfizer	2	Unknown	No
**62312**	55-59	Male	Asian	Unknown	Moderna	1	Unknown	No
**62392**	65-69	Male	White	Not Hispanic or Latino	Pfizer	7	Pre-vaccine infection	No
**A0080**	65-69	Male	White	Not Hispanic or Latino	Pfizer	8	Pre-vaccine infection	Yes
**26769**	70-74	Male	White	Not Hispanic or Latino	Pfizer	6	Pre-vaccine infection	Yes
**A0785**	70-74	Male	White	Not Hispanic or Latino	Pfizer	9	No infection	No
**A8085**	70-74	Male	White	Not Hispanic or Latino	Pfizer	6	Pre-vaccine infection	Yes
**A9893**	70-74	Male	Unknown	Unknown	Pfizer	7	Unknown	No
**A9905**	65-69	Male	Unknown	Unknown	Pfizer	8	None	No
**72155**	75-79	Male	Unknown	Unknown	Pfizer	7	None	No
**71255**	65-69	Male	Black or African American	Not Hispanic or Latino	Pfizer	6	None	No
**A3154**	70-74	Male	Asian	Not Hispanic or Latino	Moderna	8	None	No
**17763**	65-69	Male	White	Not Hispanic or Latino	Pfizer	5	None	No
**A3948**	65-69	Male	White	Not Hispanic or Latino	Pfizer	11	Unknown	No
**A2308**	65-69	Male	White	Not Hispanic or Latino	Pfizer	8	Unknown	No

Supplemental Table 3: Comparison of the demographics between PWH and PWOH

		PWH	PWOH	Statistical test
		n = 37	n = 24	
**Median Age**		68	66	ns (Mann-Whitney)
**Sex**	**Male**	100%	100%	ns (Mann-Whitney)
	**Female**	0%	0%	
**Months after the 3rd vaccine dose (median, range)**		5 (2-12)	5.7 (1-11)	ns (Mann-Whitney)
**Race/Ethnicity**	**White and Not Hispanic**	3%	72%	$p < 0.0001$ (Chi-square)
	**Black or African American and/or Hispanic**	95%	22%	
**Race**	**Black or African American**	81%	13%	
	**White**	16%	58%	
	**Asian**	3%	8%	
	**Other**	0%	4%	
	**Unknown**	0%	17%	
**Ethnicity**	**Hispanic or Latino**	19%	4%	
	**Not Hispanic or Latino**	81%	71%	
	**Unknown**	0%	25%	
